# Microstructure and Abrasive Wear Resistance of Metal Matrix Composite Coatings Deposited on Steel Grade AISI 4715 by Powder Plasma Transferred Arc Welding Part 1. Mechanical and Structural Properties of a Cobalt-Based Alloy Surface Layer Reinforced with Particles of Titanium Carbide and Synthetic Metal–Diamond Composite

**DOI:** 10.3390/ma14092382

**Published:** 2021-05-03

**Authors:** Artur Czupryński

**Affiliations:** Department of Welding Engineering, Faculty of Mechanical Engineering, Silesian University of Technology, Konarskiego 18A, 44-100 Gliwice, Poland; artur.czuprynski@polsl.pl

**Keywords:** PPTAW, cladding, deposition, abrasion, impact load, titanium carbide, synthetic metal–diamond composite

## Abstract

The article discusses test results concerning an innovative surface layer obtained using the cladding with powder plasma transferred arc welding (PPTAW) method. The above-named layer, being a metal matrix composite (MCM), is characterised by high abrasive wear resistance, resistance to pressure and impact loads, and the possibility of operation at elevated temperatures. The layer was made using powder in the form of a cobalt alloy-based composite reinforced with monocarbide TiC particles and superhard spherical particles of synthetic metal–diamond composite provided with tungsten coating. The surface layer was deposited on a sheet made of low-alloy structural steel grade AISI 4715. The layer is intended for surfaces of inserts of drilling tools used in the extraction industry. The results showed the lack of the thermal and structural decomposition of the hard layer reinforcing the matrix during the cladding process, its very high resistance to metal-mineral abrasive wear and its resistance to moderate impact loads. The abrasive wear resistance of the deposited layer with particles of TiC and synthetic metal–diamond composite was about than 140 times higher than the abrasive wear resistance of abrasion resistant heat-treated steel having a nominal hardness of 400 HBW. The use of diamond as a metal matrix reinforcement in order to increase the abrasive resistance of the PPTAW overlay layer is a new and innovative area of inquiry. There is no information related to tests concerning metal matrix surface layers reinforced with synthetic metal–diamond composite and obtained using PPTAW method.

## 1. Introduction

Processes taking place during the boring of oil and gas wells and the mining of rock in underground workings are extremely complex and difficult. Among other things, the aforesaid situation results from the mechanical properties of mined ground or rock layers and their inhomogenous geological structure (responsible for the fast wear of drilling tools used in the extractive industry). The necessity of the frequent replacement of worn-out mining blades (drills, boring crowns, cone cutters, etc.) significantly increases the costs of excavated raw materials. The properties of structural or tool materials depend both on the microstructure of the core of a given element and the condition of its surface layer. In cases of elements that do not transfer significant loads or are not exposed to intense abrasive wear during operation, the condition of the surface layer is of lesser importance. However, tools and machinery elements made of steel are exposed to abrasion combined with high unit pressure, impact loads, a corrosive environment, and high operating temperature [1,2,3,4]. In particular, the above-presented operating conditions affect tools being in direct contact with abrasives such as rock, sand, clay or other hard components present, among other things, in the ground. Globally, the aforesaid problems are present in the fossil fuels excavation industry. Power engineering and machine-building sectors compete intensively to develop modern technologies, making it possible to obtain a longer service life of tools and machinery elements used in coal mines, quarries, oil rigs and climate engineering, and during the construction of motorways. There is a high demand for spare parts of mining machinery and in particular for drilling and geological tools, which wear quickly and, consequently, lose their operational properties. Such tools have to be replaced very often, generating additional and, frequently, high costs, connected not only with the purchase or the refurbishment of new tools but, primarily, with the time needed to replace them. In addition, the dismantling and the reassembly of tools are responsible for costly downtimes. Presently, it is possible to observe a tendency of extending the service life of drilling and geological tools, even at the expense of significantly higher prices. Mining concerns find it more beneficial to buy more expensive tools characterised by higher quality than to stop production (several times) in order to retool machinery. Being a specific abrasive, the ground is not easy to define explicitly. This fact results mainly from the geological and engineering conditions of a given excavation area as well as from weather conditions present during the operation of the tool (affecting friction conditions in the ground-tool system). Abrasive wear, to which drilling and geological tools are exposed during operation in the ground, translates directly into the service life and the reliability of mining machinery. The inspection and the forecasting of tool wear in the ground prove very difficult. As of today, related engineering knowledge is limited to experimentation and the development of the so-called neural networks. The more detailed identification of the destruction of materials being in motion during extraction requires the combination of many elementary wear-related phenomena.

This issue was addressed by, among others, Kenny et al., 1976; Gharahbagh et al., 2013; Dewangan et al., 2014, 2015, Amoun et al., 2017, and Nahak et al., 2018 [5,6,7,8,9,10]. The problems encountered by the extractive industry necessitate the search for methods making it possible to reduce the wear of tools and machinery parts used in the extractive industry. Researchers and engineers constantly try to develop new ranges of tools, changing both design-related solutions and materials. However, the improvement of operational properties remains primarily connected with the improvement of the properties of the surface layer. An increase in hardness and abrasive wear resistance as well as surface processing involving the use of chemical elements improving corrosion resistance enable the extension of tool service life. It should also be noted that surface processing belongs to the most economically effective and useful methods applied in widely defined materials engineering. The making of layers characterised by new and unique properties may entirely change the operational parameters of every base material. Related publications concerning the subject discuss the obtainment of the increased abrasive wear resistance of tools primarily through the application of ceramic materials [11,12,13], diffusive carbide [14,15,16], boride coatings [17,18,19] and thermally sprayed layers [20,21,22]. Some of the above-named methods fail to produce desirable results and, in addition, are both energy-consuming and laborious. The aforesaid coatings are usually deposited on the entire surface of a given product, which is not always economically justified. Many researchers believe that the most promising technologies enabling the fabrication of abrasive wear resistant coatings should be based on high-energy density methods, including plasma or laser cladding.

Presently, in developed countries such methods are used to extend the service life of mining and drilling tools as well as to make corrosion resistant layers. The powder plasma transferred arc welding (PPTAW) or the laser metal deposition (LMD) are used by, among others, General Electric Oil&Gas and Honeywell International, i.e., leading oil and gas producers [23,24], as well as by many manufacturers of plasma arc welding systems. Various plasma arc welding methods can be applied to make surface layers using nearly any metallic material. In such cases, a deposited material becomes the primary component of the surface layer and, because of high process temperature, melts along with the substrate. Issues concerning the powder plasma transferred arc cladding process were discussed, among others, by Khaskin et al. (2016), Brunner-Schwer et al. (2018, 2019), Xia et al. (2010) and Gao et al. (2020) [25,26,27,28,29]. Recently, it has been possible to observe very high interest in the application of plasma and laser cladding processes to make composite surface layers on steels and alloys of non-ferrous metals. The above-named layers are composed of the metallic matrix reinforced with hard particles of interstitial compounds. The matrix is usually made of iron [30,31], nickel [32,33] or alloys containing the aforesaid elements. The Ni-Cr-B-Si alloy is usually applied through thermal spraying [34]. There are also numerous publications concerning the matrix containing cobalt and its alloys [31,35] (e.g., stellites [36,37]). However, stellites are less frequently referred to as matrix materials in composite layers and more often as homogenous layers. Particles reinforcing composite surface layers are various interstitial compounds, usually carbides but also nitrides and borides [15,17]. The most commonly used carbides include tungsten carbide (WC) [30,38], silicon carbide (SiC), boron carbide (B_4_C) [39] and titanium carbide (TiC) [40].

Titanium carbide (TiC) particles are widely used to reinforce structural materials (both as volume and surface reinforcement). The plasma arc melting of metallic powder and TiC particles (the granularity of which was restricted within the range of 10 μm to 14 μm) on the surface of elements made of steel AISI 304 was investigated by Bober and Grześ (2015) [41]. The use of the powder plasma transferred arc made it possible to obtain the proper joint of the deposited material components and provided appropriate adhesion to the substrate. Kindrachuk et al. (2016) [42] made a TiC-Co composite layer deposited on the surface of steel 2Cr13. The layer, composed of several variedly structured sub-layers, was applied to extend the service life of machinery elements and power generation equipment. The substrate, affected by the plasma arc, underwent self-hardening, whereas the remaining part of the layer contained the zone of molten material and the dilution zone. The structure of the layer was composed of supersaturated cobalt dendrites with dispersed TiC particles. Depending on a steel grade, it is possible to obtain its reinforcement through self-hardening. However, in cases of superalloys, the surface layer changes its chemical composition and structure (as a result of diffusion and dilution), which is an undesired phenomenon. The chromium-nickel matrix is a very popular material of the MMC composite reinforced with TiC particles. According to Onuoha (2016) [43], in the Cr-Ni alloy reinforced with TiC particles having a granularity restricted within the range of 4 μm to10 μm and 70–90 vol%, the larger grain size of the hard phase was responsible for increased abrasive wear. The mechanism of the aforesaid wear consisted primarily of micro-cutting. Sakamoto et al. (2015) [44] used 2 wt% of TiC particles to significantly improve the mechanical properties of the Cr-Ni alloy. The TiC-Co-type composite layers were also examined by Jung et al. (2015) [45]. The specimens were prepared through high-energy ball milling and liquid phase sintering. The size of the TiC particles was restricted within the range of 7 μm to 10 μm. The researchers emphasized the significance of the size of the particles of the hard phase and the type of the matrix material. It was demonstrated that fine-grained powders based on cobalt alloys inhibited the growth of TiC grains during sintering. The work does not contain any results of tribological tests.

Karantzalis et al. (2013) [46] made a cobalt alloy-based composite reinforced with TiC particles [46]. The components of the composite were melted using the vacuum arc melting method. It was found that a greater amount of the hard TiC phase favoured the grain growth in the metallic matrix. The results of the abrasive wear test proved promising. Another type of a sintered alloy, i.e., Co-TiC, was investigated by Jung et al. (2015) [45]. It was revealed that the modification of the initial powder material through the addition of cobalt nanoparticles decreased the powder sintering temperature. The obtained alloy was characterised by favourable thermal stability and advantageous mechanical properties. In their research, Anasori et al. (2016) [47] demonstrated that a magnesium alloy containing 5, 20 and 50 vol% of TiC and Ti_2_AlC sintered carbides was characterised by excellent energy absorbability. The favourable size of the particles reinforcing the matrix was restricted within the range of 5 μm to 15 μm. According to the Authors, the damping effect was obtained owing to the natural ability of materials to absorb energy, the large matrix-carbide contact area and a different thermal expansion coefficient. However, the aforesaid factors also contributed to an increased number of dislocations.

The above-presented research results concerning the making of metal matrix composite layers reinforced with TiC particles justified the formulation of the following conclusions:

- TiC is a very promising material reinforcing the matrix of metallic materials (MMC) in applications requiring high abrasive wear resistance under conditions of dry sliding friction. Matrix materials include many metals and alloys, e.g., cobalt, nickel, magnesium and aluminum;
- All tests were based on the application of various combinations of the hard reinforcing phase and the metal matrix;
- Abrasive wear resistance tests of Me-TiC-type composite layers were primarily performed at room temperature and under conditions of dry sliding friction;
- In most tests, the composite material was obtained using sintering methods, where the volume fraction of carbides was restricted within the range of 40% to 60%. The microstructural tests revealed the proper dilution and the uniform distribution of alloying elements and carbide particles in the matrix material;
- Depending on the type of the metal matrix and test conditions, the effect of the high content of TiC in the composite can be both favourable [42] and disadvantageous [48]. However, it should be noted that reinforcing the metal matrix with TiC particles favourably reduces abrasive wear regardless of the type of the matrix and the reinforcement-matrix ratio in the composite.

The analysis of related reference publications and the results of individual research led to the conclusion that it was possible to obtain a composite surface layer in the ceramic reinforcement–metal matrix system of phases, the microstructure and abrasive wear resistance of which would be similar to those of sintered carbides [49].

## 2. Materials and Methods

### 2.1. Materials

**Figure 1:**
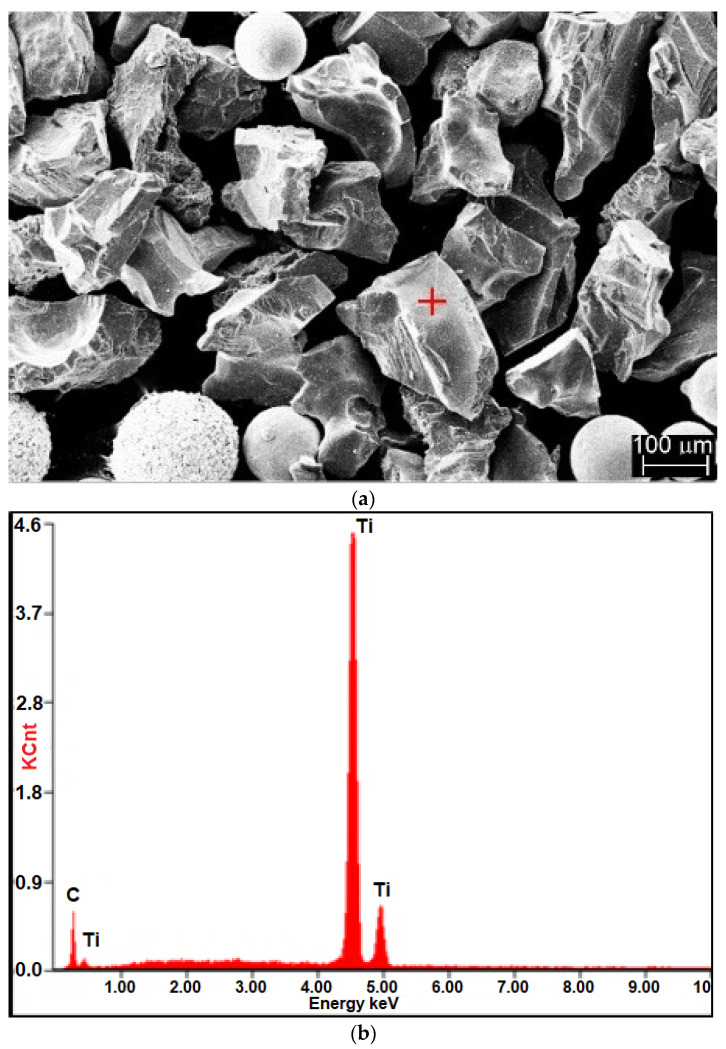

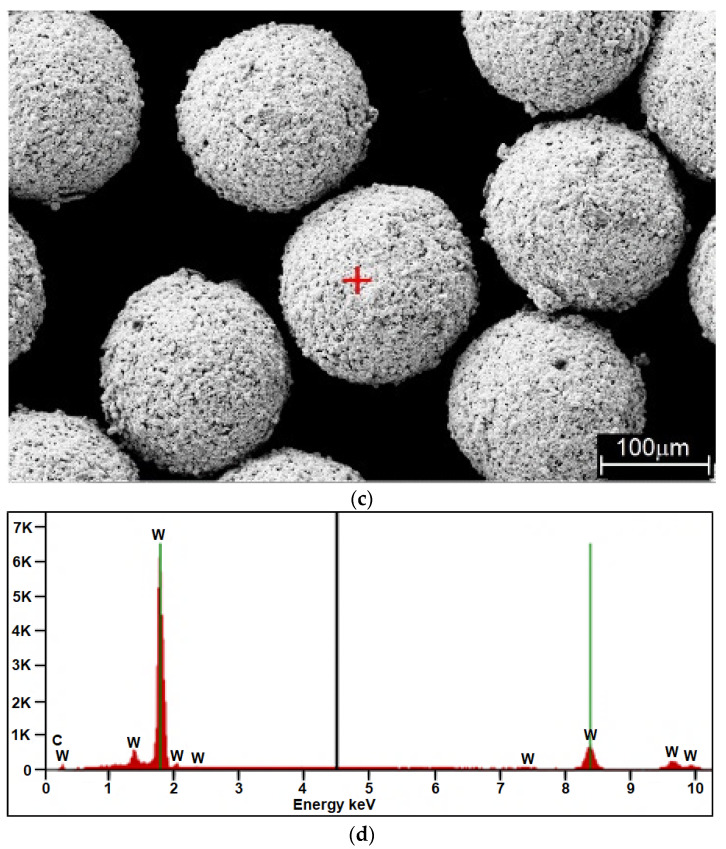
Components of the hard phase in the Co3+TiC+PD-W powder: (**a**) sharp-edged titanium carbide, TiC (mag. ×300); and (**c**) synthetic metal–diamond composite in the tungsten lagging, PD-W (mag. ×500), and diagram of the energy of scattered X-radiation with energy lines present in the area of components: (chemical elements (ceramic particle of TiC and metal matrix)) subjected to analysis (**b**) ceramic particle of TiC; (**d**) tungsten-coated synthetic metal–diamond composite (PD-W).

The surface layer was deposited using the powder plasma transferred arc welding (PPTAW) method on specimens having dimensions of 75 mm × 25 mm × 10 mm, made of low-alloy structural steel grade AISI 4715 (Table 1). The cladding process was performed using metal-matrix composite (MMC) powder belonging to the group of Co3 alloys (in accordance with EN 147000:2014) [50]. The powder contained superhard phases in the form of ceramic particles made of crushed sharp-edged titanium carbide (TiC) (see Table 2, Figure 1a) and spherical particles made of synthetic metal–diamond composite sinter in the tungsten lagging (PD-W) (Harmony Industry Diamond, Zhengzhou, China) (see Table 2, Figure 1b). The components of the powder were mixed in a Turbula T2F laboratory powder mixer-shaker (Glen Mills Inc., Clifton, NY, USA) using ceramic balls.

### 2.2. Plasma Processing

**Figure 2:**
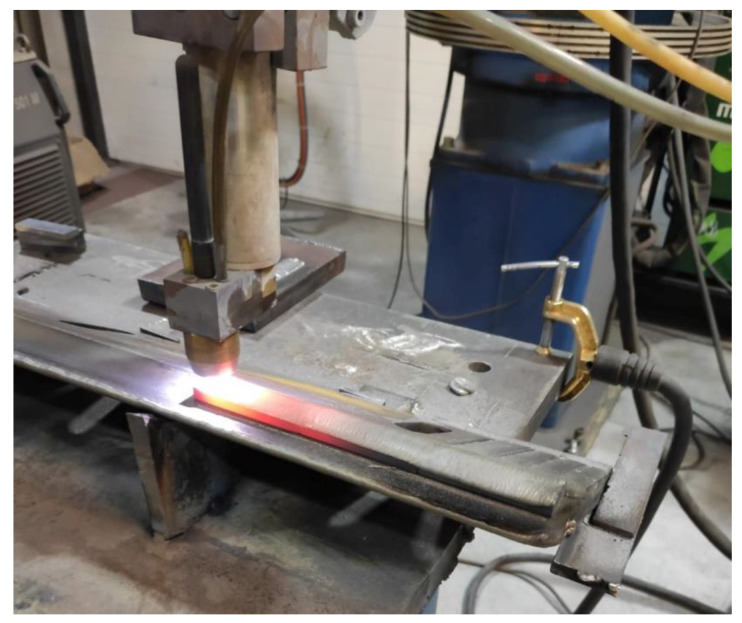
View of the powder plasma transferred arc welding (PPTAW) processes.

The plasma deposition process was carried out with surfacing machine Durweld 300T PTA with a maximal current of about 300A. For experiment, the machine powder plasma surfacing torch PT 300AUT (Durum Verschleiss-Schutz GmbH, Willich, Germany) with a thoriated tungsten cathode 4 mm in diameter (Figure 2) was used, mounted on industrial robot Fanuc R-2000iB (FANUC Ltd., Oshino-mura, Japan) arm.

PTA welding system Durweld 300T PTA was PLC-controlled and equipped with a HMI-interface, gas mass flow meter and powerful water cooling unit. PLC provides reliable operation and allows for easy integration in robot cells. The powder cladding system consisted of a computer-controlled powder feeding system PFU 4 (4th generation Powder Feeding Unit design) and a PTA torch integrated with a six-axis robot. Powder feeder was intended for applications that require feeding of different powders in the weld pool, i.e., matrix and carbides. Feeding rate step was controlled via feeding wheel speed directly from PLC. The coaxial injection of the powder was performed using the plasma, carrier and shielding gas. The cladding process was performed using the following gas flow rates: plasma gas (Ar) = 1.6 L/min, carrier gas (Ar + 5% H_2_) = 4 L/min and shielding gas (Ar + 5% H_2_) = 12 L/min.

The determination of the optimum range of cladding parameters required the making of a series of weave-bead claddings using a main arc current of 40, 60, 80, 100 and 120 A; a cladding speed restricted within the range of 1 mm/s to 4 mm/s; and a powder feed rate restricted within the range of 10 g/min to 30 g/min. The optimal processing parameters for robotic plasma cladding were established based on NDT and metallographic tests. The cladding parameters identified as optimum (Table 3) were those ensuring the uniform distribution of the powder over the entire liquid metal area in the melt pool, the proper depth of penetration g = 1.2 mm, layer height h = 3 mm and the dilution of the base material in the cladding amounting to D = 4.5%.

### 2.3. Testing Methodology

The analysis of the morphology and the size of the composite powder (MMC) was based on images obtained using a scanning electron microscope. The assessment of the quality of the layer and the detection of cladding imperfections (if any) such as cracks, porosity, spikes, undercuts, and shape and dimension-related imperfections required the performance of non-destructive tests, including visual tests (VT), penetrant tests (PT) and tests concerning the roughness (Ra) of the deposited layer. The assessment of surface properties was based on the analysis of macro and microscopic metallographic test results, chemical composition analysis, X-ray diffraction results, hardness and roughness measurement results, and results of tests concerning metal-mineral abrasive wear resistance and impact resistance.

#### 2.3.1. Composite Powder Morphology, and the Structure and Chemical Composition of the Deposited Layer

The assessment of the surface and the size of the particles of the composite powder as well as of the structure of the deposited layer were performed using a Zeiss Supra 25 scanning electron microscope (Carl Zeiss AG, Oberkochen, Germany). The tests were performed using a detector of secondary electrons (SE), an accelerating voltage of 20 kV and a probe current of 5 nA. The chemical composition of the powder components and of the deposited layer was identified on the basis of tests performed using a Zeiss Supra 25 scanning electron microscope featuring an EDS and UltraDry EDS detector (for X-ray microanalysis) (ThermoFisher Scientific, Waltham, MA, USA). The tests were performed on the surface of the specimens using a point or an area-based analysis.

**Figure 3:**
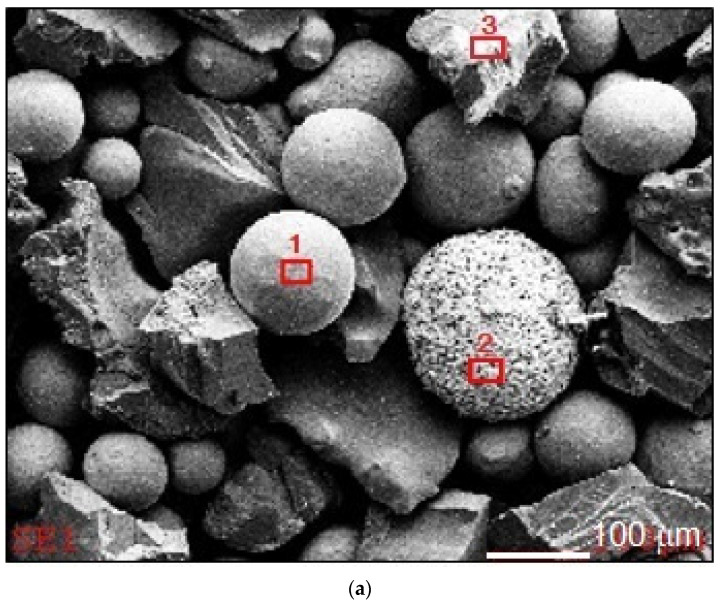

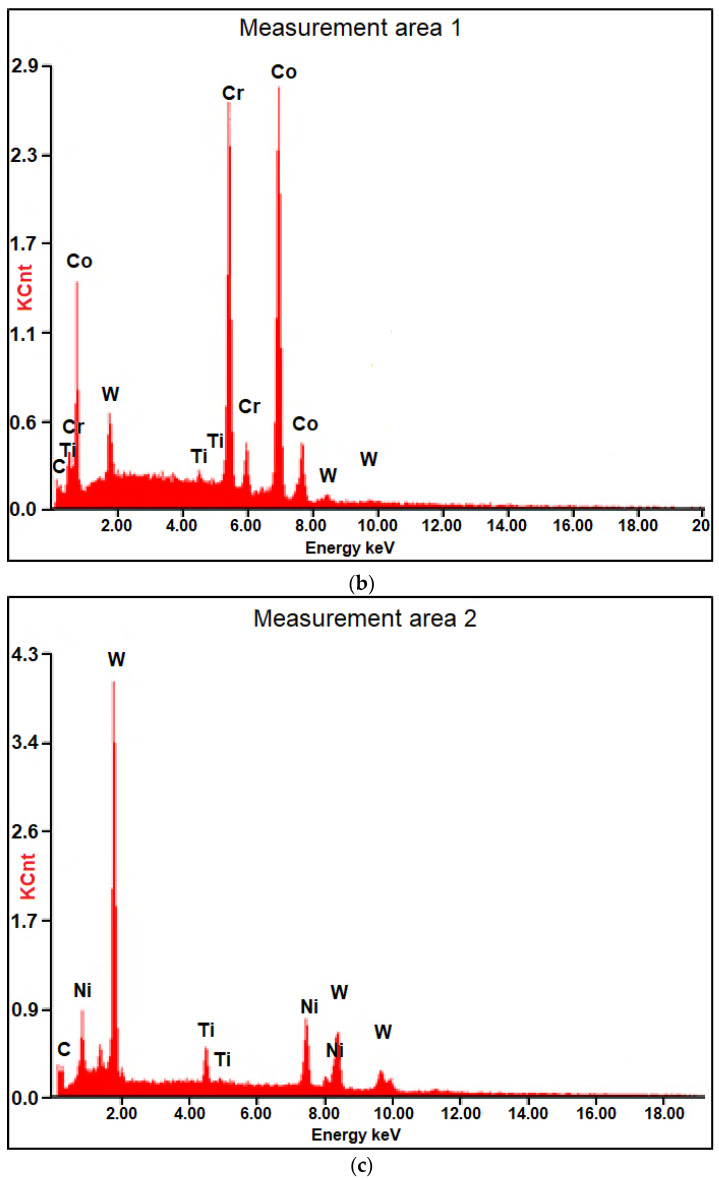

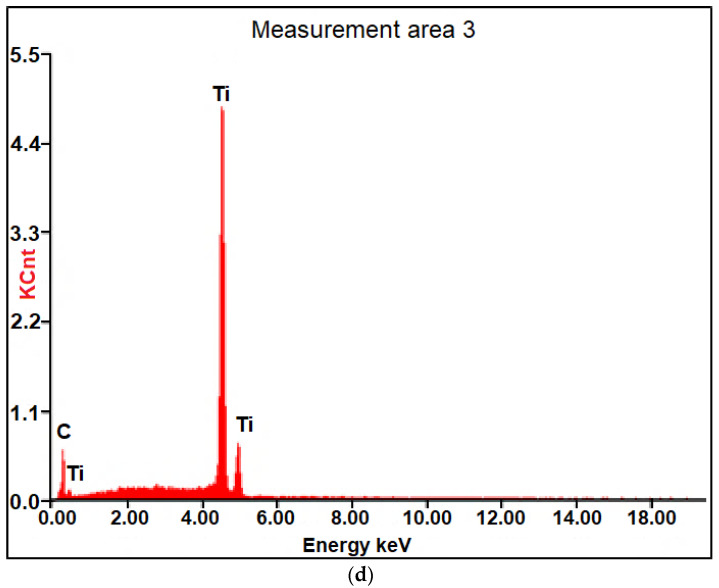
Results of the microanalysis of the chemical composition of the Co3+TiC+PD-W composite powder: (**a**) SEM image of the morphology of the powder particles with the area subjected to analysis and the diagrams of scattered X-radiation with energy lines present in the area of components (chemical elements) subjected to analysis: (**b**) matrix, (**c**) ceramic particle (TiC) and (**d**) tungsten-coated synthetic metal–diamond composite (PD-W).

Results of composite powder (Co3+TiC+PD-W) morphology tests are presented (in the form of the SEM images and diagrams of scattered X-radiation) in Figure 3. The tests revealed that the size of the powder particles was restricted within the range of 60 μm and 250 μm (mediana Q_50_ = 152 µm) and constituted the mixture of irregular and spherical components. The tests were performed on the surface of the powder particles using point or micro-area-based analysis.

The mixer-shaker used for the mixing of the components of the Co3+TiC+PD-W composite powder enabled the making of the homogenous composition of the composite material. The use of an additional element facilitating the stirring process (i.e., ceramic balls) precluded the segregation of the components and the formation of larger agglomerates of the plastic phase. Methods used previously to mix the powder components, e.g., in a conical mixer, failed to produce desirable results.

#### 2.3.2. Non-Destructive Tests

**Figure 4:**
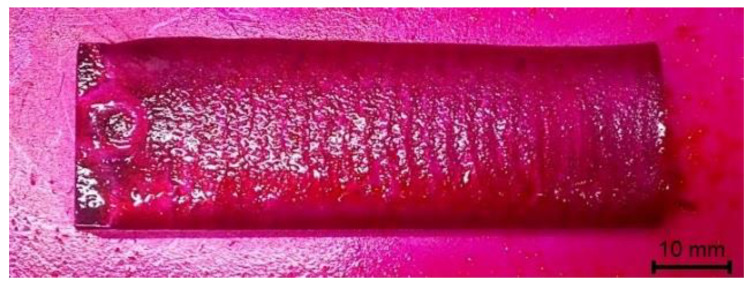
View of the sample during the penetrant testing (PT).

The visual tests (external visual inspection) and the penetrant tests were performed in accordance with the requirements specified in related standards, i.e., ISO 17637 [51] and ISO 3452-2, respectively [52]. The visual tests involved the verification and the assessment of the condition of the deposited layer by the unaided eye (direct visual test). Before the test, the surface to be inspected was subjected to thorough cleaning and drying. The penetrant tests were performed using a system of dye penetrants (System Designation Type II, Sensitivity 2) Cd-2 PT ISO 3452-2 II Cd-2 and EN 571-1 (Figure 4). The surface roughness measurements were performed in five areas of the deposited layer, using a Surtronic 3+ surface roughness tester (Ametek Taylor Hobson, Berwyn, PA, USA).

#### 2.3.3. Metallographic Examination and X-ray Diffraction Analysis

The microscopic tests were performed using metallographic specimens subjected to standard preparation. The etchant was the so-called “aqua regia”, i.e., the 3:1 mixture of concentrated hydrochloric acid and nitric acid; the time of etching was determined experimentally. The observation and the recording of macro and microstructural images were performed using an Olypmus SZX9 stereoscopic microscope (Olympus Corporation, Tokyo, Japan) equipped with a Moticam 5.0+ digital camera and a Motic Images plus 3.0 software programme as well as an Olypmus GX 71 inverted metallographic microscope (Olympus Corporation, Tokyo, Japan). The phase composition of the deposited layer was determined on the basis of X-ray diffraction analysis performed using a Panalytical X’Pert Pro MPD diffractometer (Malvern Panalytical Ltd., Malvern, UK) and the filtered radiation (filter Kβ Fe) of a cobalt anode lamp (λKα = 0.179 nm). The diffraction patterns were recorded in the Bragg–Brentano geometry, using a PIXcell 3D detector and the axis of the beam deflected within the angle range of 20 to 110 (2θ) (increment = 0.05°, counting time per increment = 100 s). The diffraction patterns were subjected to analysis involving the use of a dedicated Panalytical High Score Plus software programme and a PAN-ICSD structural database. The X-ray quantitative phase analysis was performed using the Rietveld refinement method.

#### 2.3.4. Density and Porosity of the Deposited Layer

**Figure 5:**
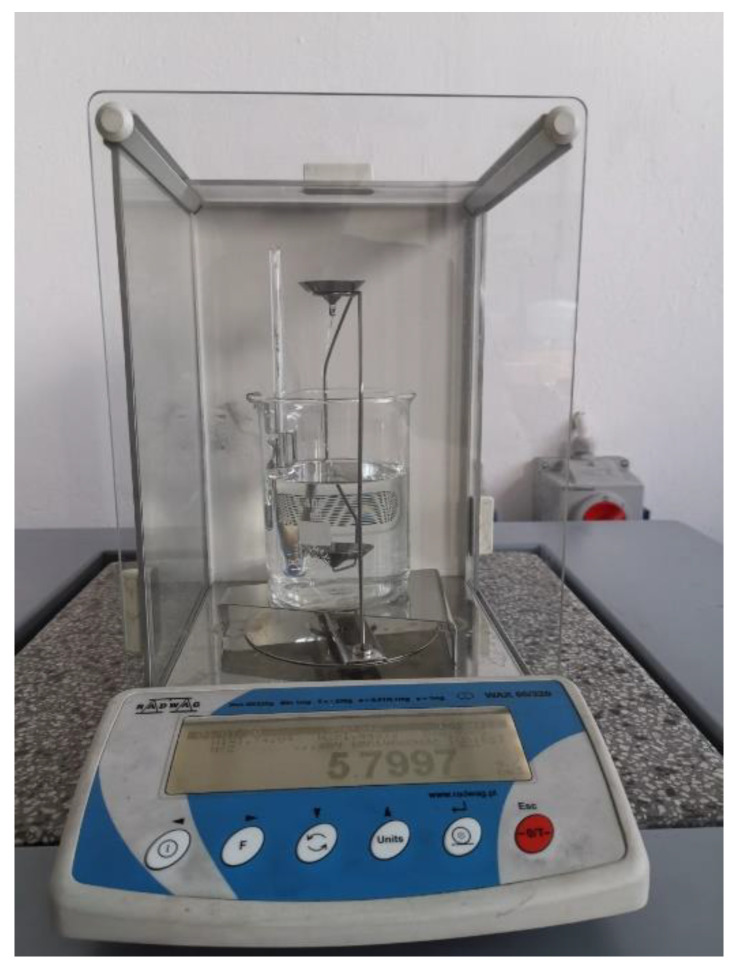
View of an analytical balance for density of the deposited layer measurement.

The density of the surface layer was measured using the Archimedes method in accordance with the ISO ASTM D792 standard [53]. The measurement involved the use of a Radwag AS 220.R2 analytical laboratory balance (Radwag, Warsaw, Poland) along with a set for Archimedes-method-based density measurements (Figure 5).

The analysis of the surface layer roughness degree was performed using a µCT Nanotom 180N micro-tomograph (Ge Sensing & Inspection Technologies GmbH, Wunstorf, Germany) equipped with an X-ray tube having a maximum voltage of 180 kV. Tomographic images were recorded using a Hamamastu 2300 × 2300 pixel decoder. The virtual reconstruction of tested objects was performed using a GE datosX ver.2.1.00 software programme. All of the tomographic images were made using a source voltage of 140 kV and 200 µA; the element was rotated by 360°, in 2400 steps. The time of exposure amounted to 500 ms, exposure averaging amounted to 3, the image refresh rate amounted to 1 and the time of scanning a single element was *t* = 80 min. The analysis of roughness was performed using an MyVGL programme software.

#### 2.3.5. Hardness Measurements

**Figure 6:**
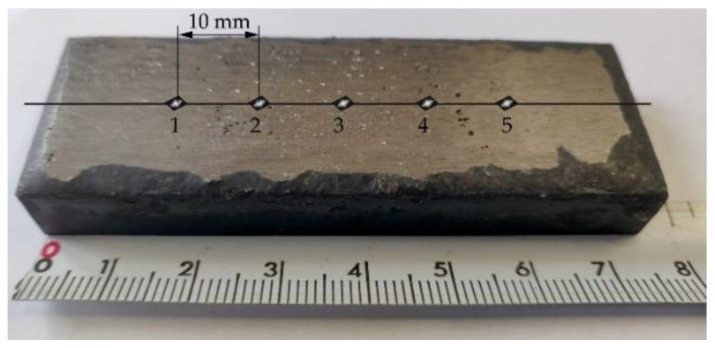
Exemplary locations of measurement points on the surface of the wear resistant layer.

The hardness measurements concerning the external surface and the cross-section of the surface layer and of the reference material (abrasion-resistant heat-treated steel having a nominal hardness of 400 HBW) were performed using the Vickers hardness test (in accordance with the procedure referred to in the ISO 6507-1 standard) [54] and a Future-Tech FM-ARS 9000 hardness tester with an automatic measurement line and an image analysis system (Future-Tech Corp., Kawasaki, Japan). The specimen surface hardness was determined within the HV10 scale using a test load of 10 kgf (total test force 98 N) and time *t* = 30 s. The hardness tests were performed at five test points on the surface layer subjected to grinding. Exemplary locations of measurement points on the surface of the abrasive wear resistant layer are presented in Figure 6.

The microhardness testing was done using Vickers method HV0.5. The hardness measurements concerning the cross-section of the surface layer were performed on metallographic specimens, at 10 measurement points, separately for the particles of the hard phase and the matrix.

#### 2.3.6. Abrasive Wear Resistance Tests

**Figure 7:**
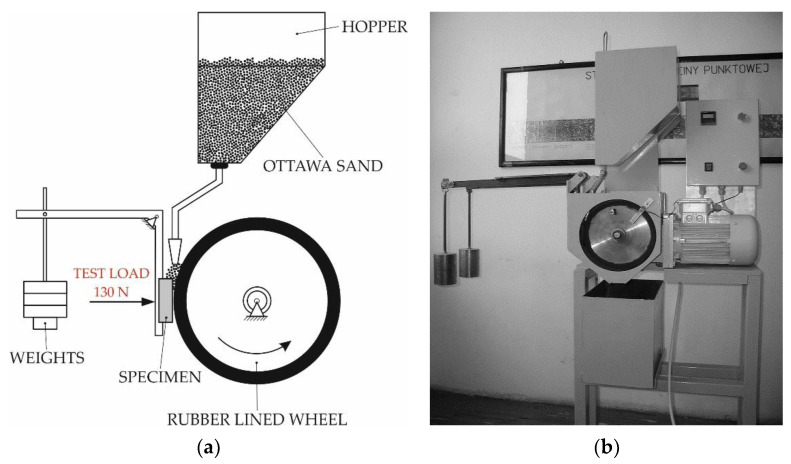
Testing station for metal-mineral abrasive wear resistance tests in performed in accordance with ASTM G65: (**a**) schematic diagram and (**b**) main view [30].

The metal-mineral abrasive wear resistance test of the surface layer and of the reference material (abrasion-resistant steel grade AR400) was performed in accordance with ASTM G 65-00, Procedure A [55], using the, “rubber wheel” machine (Figure 7).

The “rubber wheel” abrasive wear test governed by the ASTM G65 standard is the most popular test used in materials engineering to assess metal-mineral abrasive wear resistance. The abrasive used in the test was quartz sand, the grain size of which was restricted within the range of 50 mesh to 70 mesh (0.297–0.210 mm); the sand was fed gravitationally to the friction zone. The experimental tests concerning the deposited layer and the reference material involved the preparation of two specimens having dimensions of 75 mm × 25 mm × 10 mm. During an approximately 30-min-long test, the rubber wheel made 6000 revolutions. The test material was subjected to a pressure force of 130 N, whereas the feed rate of the abrasive (A. F. S. Testing Stand 50–70 mesh) amounted to 335 g/min.

Before and after the abrasive wear test, the specimens were weighed on the laboratory balance with an accuracy of up to 0.0001 g. The average density of the deposited layer and that of the reference material was determined on the basis of three measurements of the specimen density, sampled and weighed at room temperature in air and liquid. The volume loss was calculated on the basis of the measured average density of the deposited surface layer and the average specimen mass loss after abrasion, using the following formula (1):

(1) $$\mathrm{volume}\;\mathrm{loss}\;\left[{\mathrm{mm}}^{3}\right]=\frac{\mathrm{mass}\;\mathrm{loss}\;\left[g\right]\;}{\mathrm{density}\;\left[\frac{g}{{\mathrm{cm}}^{3}}\right]}\times 1000$$

The specimen surface abrasion area was subjected to microscopic examination performed using a Zeiss Smartproof 5 confocal microscope (Carl Zeiss AG, Oberkochen, Germany). The type of abrasive wear was identified using a criterion based on the quotient of the cross-sectional areas of the sum of the two-sided upsetting of the material near micro-scratch F1 and the depth of micro-scratch F2.

The material loss in the surface layer during abrasive wear was classified in relation to micro-ridging, i.e., the plastic deformation of contact areas and the upsetting of the material on both sides of the micro-ridge, where F1/F2 = 1; micro-cutting, where F1/F2 = 0; and micro-scratching, if the material was partly deformed plastically and partly cut in the form of chips as wear products, where 0 ≤ F1/F2 ≤ 1 [56].

#### 2.3.7. Impact Resistance Tests

**Figure 8:**
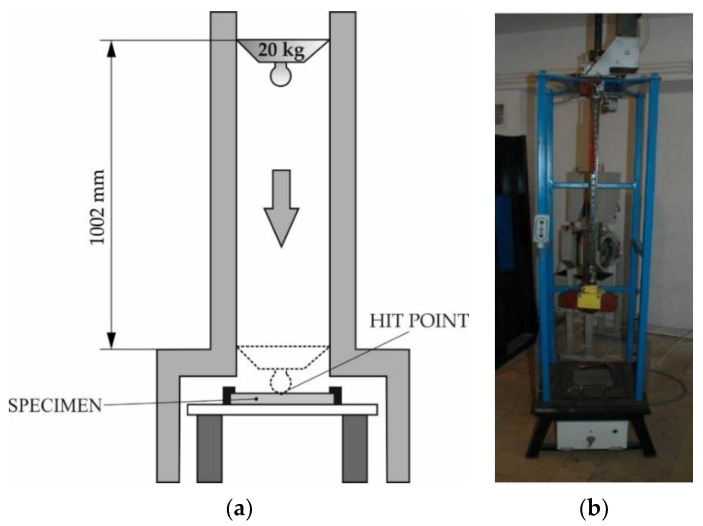
Test rig used in the tests of the impact resistance of the surface layer: (**a**) schematic diagram and (**b**) main view [21].

The impact resistance tests of the deposited layer were performed in laboratory conditions using a dedicated testing station (Figure 8). The tests involved the use of a specimen previously subjected to penetrant testing (aimed to identify already existing cracks and surface imperfections). A criterion adopted in the impact resistance test was the number of cracks and chips as well as the general comparative assessment of damage caused by the multiple strokes of the deposited layer with a 20 kg tool (ram) released freely from a height of 1.02 m (impact energy of 200 J). The condition of the deposited layer was identified on the basis of visual tests after 5, 10 and 20 strokes.

## 3. Results and Discussion

### 3.1. Non-Destructive Tests–Visual Test Results

**Figure 9:**
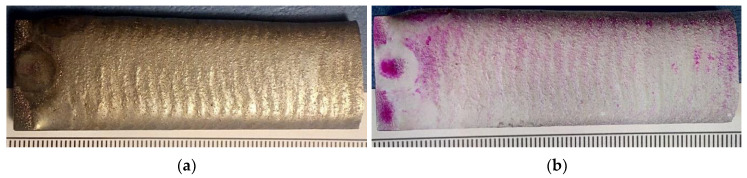
Surface layer made using the Co3+TiC+PD-W composite powder: (**a**) layer after the visual tests (VT) and (**b**) layer after the penetrant tests (PT).

The visual and penetrant tests of the surface layer only revealed the presence of welding imperfections in the form of shallow crater at the end of weld pass (Figure 9b). The above-named tests did not reveal such imperfections as cracks, porosity, spikes, undercuts, spatter, or shape and dimension-related imperfections. The deposited layer was characterised by relatively low roughness (average value *Ra* = 14 μm), uniform surface and the symmetric overlapping of successive cladding beads (Figure 9a). According to Przestacki et al. (2014) [57], surface roughness parameter (*Ra*) of deposits of tungsten carbide surface layer obtained using the cladding with Direct Laser Deposition (DLD) method is up to 32 μm. Additionally, cobalt matrix composite coatings obtained by thermal spraying can present a high surface roughness *Ra*, often significantly exceeding 30 µm [58]. PPTAW cladding with a weaving bead trajectory, at a relatively high value of the welding current (80A), resulted in a good thermal activation of the hard reinforcing phase wetting process by the liquid matrix metal. The composite layer formed correctly with a relatively low surface roughness, despite the rather unfavourable morphology of the TiC grains, which had an irregular shape and a very expanded surface.

Because of the fact that the cladding process is allied to welding and qualified as special, it is necessary to subject a given cladding technology to verification based on adopted standards, e.g., ISO 15614-7 [59]. However, the application of the above-named standard in relation to deposited composite layers may prove problematic. The aforesaid composite layers could pose a problem in terms of inspection in accordance with quality standards and standard-related quality levels as the requirements specified in the standards preclude the acceptance of surface elements containing imperfections. On the other hand, it should be noted that welding imperfections, for example, in the form of cracks contribute to the reduction of stresses in elements subjected to cladding and, consequently, improve the greasing conditions of the interacting surfaces of friction association. With respect to the special application of a given product, the transverse crack in the composite layer can be considered as acceptable. Powder plasma surfacing enabled the formation of surfaced layer with quality level B. According to ISO 5817 [60] norm, level B corresponds to the highest quality of manufactured layers.

### 3.2. Metallographic Test Results and the Results of XRD Analysis

**Figure 10:**
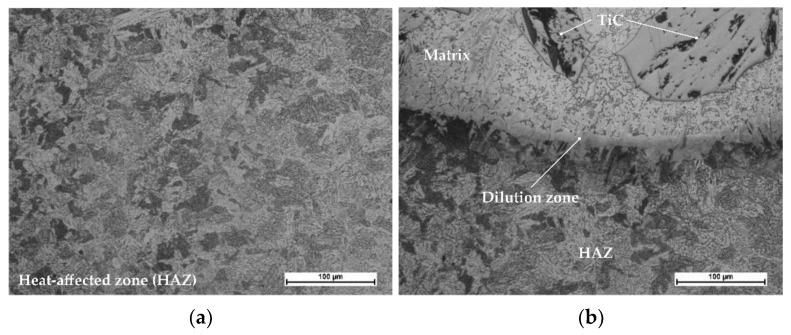

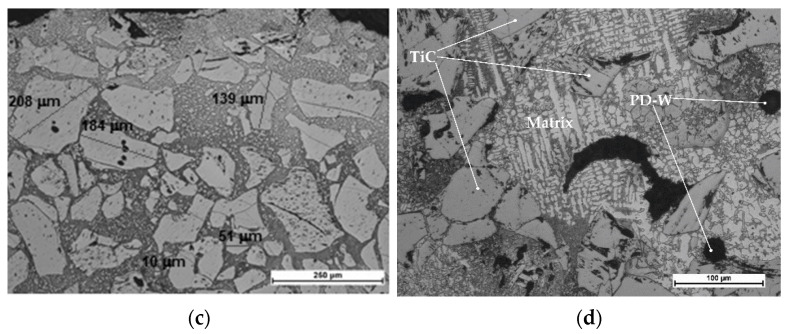
Microstructure of the surface layer (PPTAW metal deposition method, Co3+TiC+PD-W composite powder) deposited on structural low-alloy steel AISI 4715: (**a**) heat affected zone (HAZ), (**b**) dilution zone, (**c**) size and distribution of the hard phase in the middle of the cladding and (**d**) structure of the matrix of the solid solution near the padding weld.

**Figure 11:**
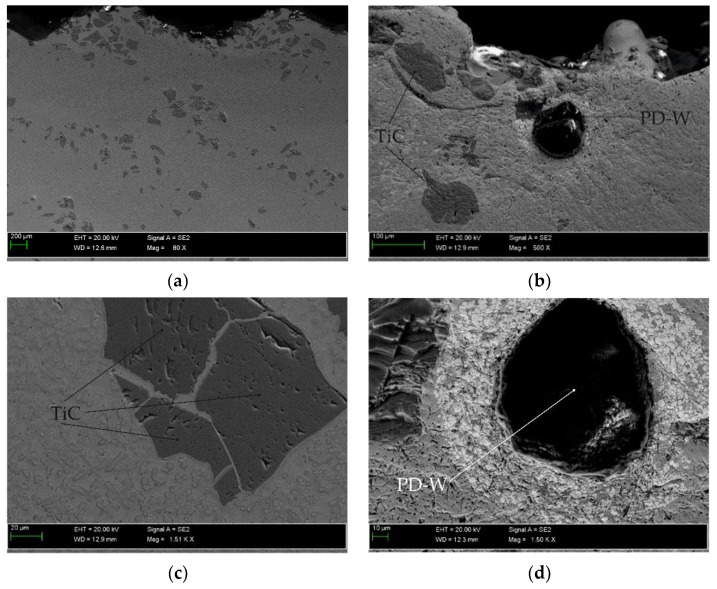
Microscopic image (SEM) of the surface layer (PPTAW metal deposition method, Co3+TiC+PD-W composite powder) deposited on structural low-alloy steel AISI 4715: (**a**) distribution of ceramic particles in the matrix, (**b**) agglomerate of ceramic particles of crushed sharp-edged titanium carbide (TiC) and the spherical particle of the synthetic metal–diamond composite (PD-W), (**c**) single particle of titanium carbide (TiC) and (**d**) the single particle of the synthetic metal–diamond composite (PD-W) in the lagging of tungsten that partly passed to the solid solution of the cobalt alloy.

**Figure 12:**
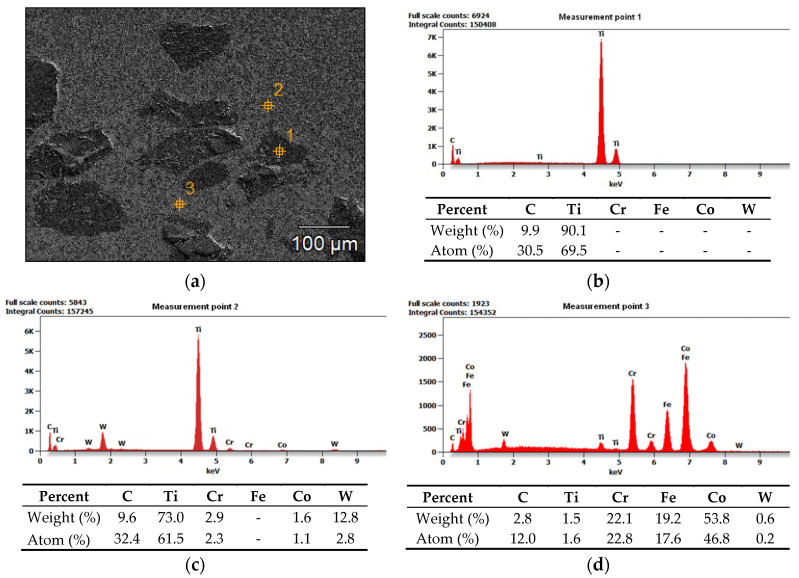
Results of the microanalysis of the chemical composition of the surface layer (PPTAW metal deposition method, Co3+TiC+PD-W composite powder) deposited on structural low-alloy steel AISI 4715: (**a**) area subjected to analysis, (**b**–**d**) diagram of the energy of scattered X-radiation with energy lines present in the area of components (chemical elements (ceramic particle of TiC and metal matrix)) subjected to analysis.

The results of the microscopic metallographic observations made it possible to identify the structure of the matrix as well as the type, distribution and dimensions of the surface layer reinforcement. The observations were performed using magnification restricted within the range of 50 times to 500 times. The observations were concerned with the subsurface as well as the middle zone and the dilution zone of the cladding and of the HAZ of the substrate. The results of the microstructural observations are presented in Figure 10. The SEM photographs are presented in Figure 11. The SEM observations were performed using a magnification of 80, 500 and 1500 times. The results concerning the microanalysis of the chemical composition of the deposited layer are presented in Figure 12.

The tests involving the use of light microscopy revealed that the microstructure of the metal matrix of the surface layer was dendritic, multi-directional and contained numerous inclusions of ceramic particles of titanium carbide (TiC) as well as single particles of the synthetic metal–diamond composite. The subsurface zone of the cladding contained agglomerates of hard phase particles characterised by smaller dimensions, whereas the dilution zone contained relatively larger particles. Titanium carbide (TiC) is characterised by favourable mechanical properties, yet, similar to most ceramic materials, it is also known for its brittleness. Plasticity limited by strong bonds translates into susceptibility go catastrophic cracking. Reference publications do not provide information concerning the crack resistance of titanium carbides or nitrides. It is probable that the first phase of the cooling of the liquid metal in the melt pool is accompanied by the cracking of the carbide, triggered by the concentration of tensile stresses in carbide defects (Figure 11c). Tests concerning tensile stresses generated during the laser-aided alloying of steel with cobalt alloys (using various laser process parameters) are discussed, among others, in publications [61,62]. As regards the particles of synthetic metal–diamond composite coated with tungsten, only some part of tungsten passes to the solution (Figure 12d). The remaining amount of tungsten reacts with diamond particles and creates the coating with tungsten carbide (WC), characterised by favourable thermal stability and abrasive wear resistance. In addition, even the thin layer of the tungsten coating provides the cohesion of the particle, increases the strength of the diamond and improves the thermal conductivity of the alloy (thus extending the service life of drilling tools). Many properties of cobalt alloys result from the crystalline structure of this chemical element. Alloying agents such as Ni, Fe and C stabilise regular face-centred structure A1 of cobalt, which, above a temperature of 417 °C, transforms into crystals of hexagonal close-packed lattice A3 stabilised by Cr, W and Mo. At ambient temperature, cobalt alloys, instead of the hexagonal phase, often contain the metastable regular face-centred phase. The above-named phase, referred to as cobalt (alloy) austenite, is a solid solution: Cr, Ni, Fe, W, Mo or Mn in cobalt [63].

Because of the dilution of the weld metal in the layer, the chemical composition of the layer differed from the chemical compositions of the powders used in the cladding process. The composite powder contained up to 5% Fe, whereas the layer contained the increased amount of this chemical element (Fe = 19.2%, see Figure 12d). The passage of Fe from the base material to the weld deposit was affected by the method and the parameters of the cladding process. The microstructure of the matrix was chemically inhomogenous. The dendritic area was formed of cobalt austenite, solution-hardened with chromium, tungsten or molybdenum. The interdendritic eutectics were rich in chromium, tungsten, silicon and carbides. In the designed alloy, Cr was “tasked with” providing corrosion resistance and reinforcing the solid solution by forming M_7_C_3_ and M_23_C_6_ carbides. According to Madadi et al. (2011) [64], the significant amount of tungsten in the layer (amounting up to 13%) can reinforce the solid solution and favour the formation of MC and M_6_C carbides as well as intermetallic phases.

**Figure 13:**
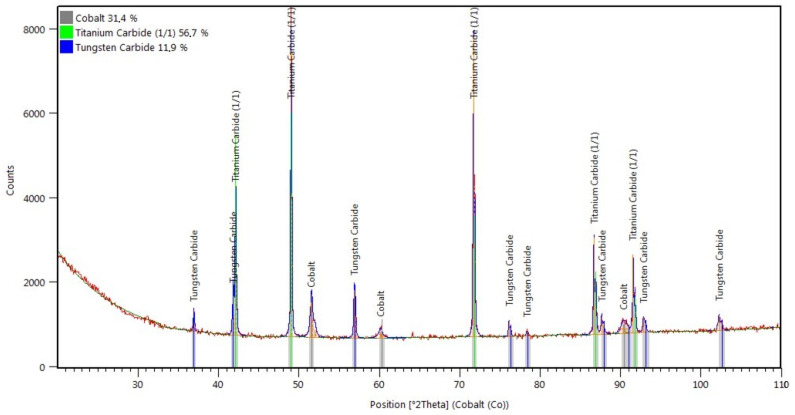
XRD pattern of the surface layer (PPTAW metal deposition method, Co3+TiC+PD-W composite powder) deposited on structural low-alloy steel AISI 4715 with the standard lines of identified crystalline phases.

The X-ray diffraction phase and quantitative analysis were performed to identify phases present in the layer. The XRD pattern is presented in Figure 13, whereas the results of the X-ray qualitative phase analysis are presented in Table 4. The analysis revealed the presence of approximately 31% of γ-Co (cobalt austenite), nearly 57% of regularly-structured titanium carbide (TiC) and nearly 12% of hexagonal tungsten carbide (WC).

### 3.3. Deposited Layer Density and Porosity

The performance of calculations concerning the specific density of the composite and the parameters related to its porosity and absorbability involved the sampling of the surface layer for test specimens. The results of related measurements and calculations are presented in Table 5.

The composite layer was not porosity-free. The total porosity of the layer amounted to approximately 6%. In spite of the foregoing, the layer appears promising as regards further tests performed on an in situ basis using a drilling tool. Owing to its slight porosity, the layer can keep lubricant in its pores and thus reduce friction between interacting surfaces.

### 3.4. Hardness Test Results

**Figure 14:**
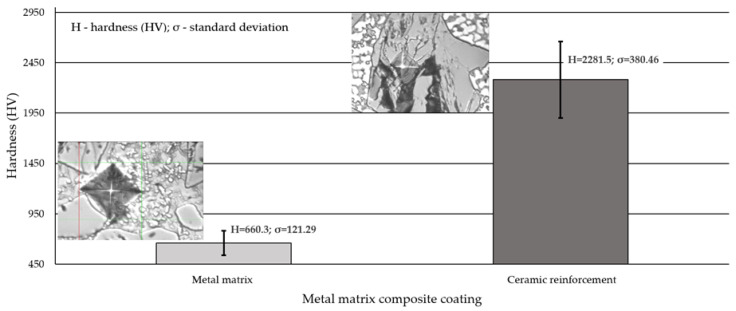
Hardness (HV0.5) measurement results concerning the cross-section of the deposited layer (PPTAW metal deposition method, Co3+TiC+PD-W composite powder) deposited on structural low-alloy steel AISI 4715.

The results of the hardness measurements concerning the external surface and the cross-section of the surface layer are presented in Table 6 and Figure 14, respectively.

The hardness of the surface layer over the entire external area was relatively uniform and, on the average, amounted to approximately 689 HV10 (59.7 HRC). In turn, the hardness of the reference material used in the abrasive wear resistance tests (abrasion-resistant steel AR400) was lower by more than 265 HV10. The hardness measurements involving the cross-section of the surface layer revealed that the average microhardness of the cobalt alloy-based interdendritic areas amounted to 660 HV0.5 and was lower by approximately 4% than the hardness measured on the surface of the deposited layer. The results concerning the microhardness of the tested layer revealed that the microhardness increased along with the distance between the measurement point and the fusion line (growing towards the surface of the layer). As is known, the microstructure of the layer in the area adjacent to the HAZ differed from the microstructure of the surface layer of the cladding as regards the cladding crystallisation manner [65]. The higher hardness could also result from the significant amount of the hard phase particles near the surface of the cladding. The average hardness of the particles of the hard phase reinforcing the matrix exceeded 2280 HV0.5. It should also be noted that the measurements concerning the hardness of titanium carbide (TiC) did not pose difficulties, yet the measurements concerning the hardness of the synthetic metal–diamond composite appeared problematic. The measurements results confirmed the effect of the base material on microhardness and reflected the chemical and structural homogeneity of the deposited layer.

### 3.5. Abrasive Wear Test Results

**Figure 15:**
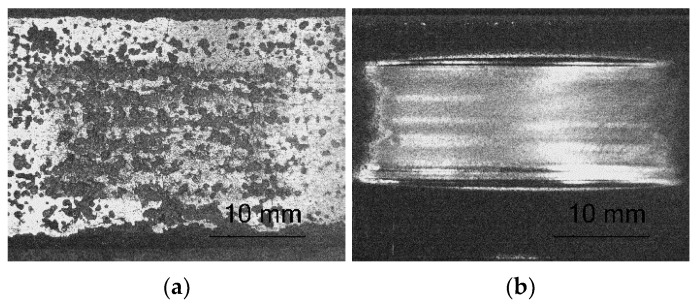
Surface of the composite layer and the surface of abrasion-resistant steel AR400 after the metal-mineral abrasive wear resistance test ASTM G64: (**a**) magnified area of layer abrasion and (**b**) magnified area of steel abrasion after the abrasive wear resistance test.

**Figure 16:**
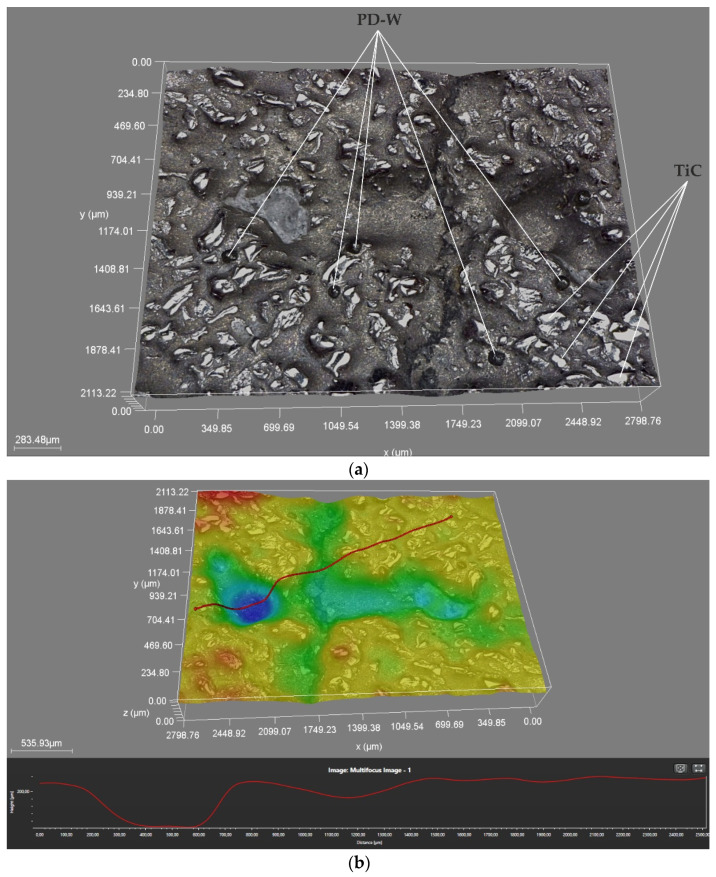

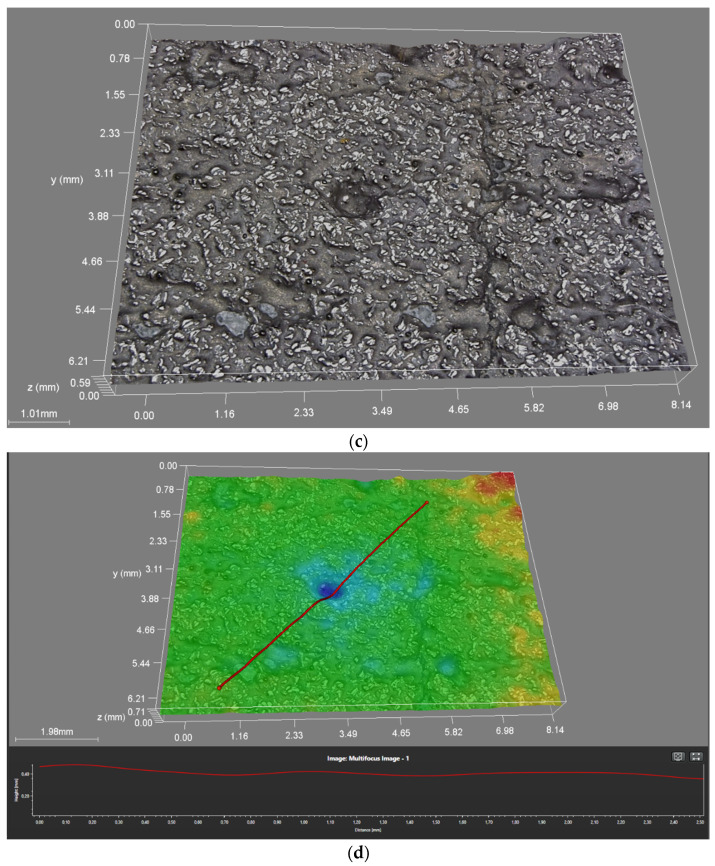
Surface of the composite layer after the metal-mineral abrasive wear resistance test observed using the confocal microscope: (**a**,**c**) main view of specimen wear, (**b**,**d**) measurement of defects (single craters).

The tests concerning the metal-mineral abrasive wear resistance of the surface layer referred to the abrasive wear resistance of the plate made of popular abrasion-resistant steel AR400 (made by a Swedish manufacturer). As a result, it was possible to determine the relative abrasive wear of the surface layer (Table 7). The nature of the abrasive wear of the surface layer was assessed on the basis of visual tests (Figure 15) and observations involving the use of a confocal microscope (Figure 16).

The intensity of abrasive wear affecting the surface layer was medium as, after interaction with the layer, the grains of sand were partly crushed. As expected, the relative abrasive wear resistance of the deposited layer was higher than that of abrasion-resistant steel AR 400 (almost 140 times). The loss of mass after the test amounted to a mere 0.0093 g. The analysis of the surface condition after test ASTM G65, Procedure A revealed the abrasive mechanism of wear. The hard phase, in the form of titanium carbide (TiC) and the particles of the synthetic metal–diamond composite uniformly distributed in the cobalt matrix, provided a natural barrier to the abrasive. The dominant wear mechanism affecting the surface layer was micro-cutting manifested by continuous micro-scratches and, to a significantly lesser extent, micro-ridging. The grains of the abrasive created slight micro-scratches on the surface of the composite layer. Locally, the course of the micro-scratches deviated from the rectilinear direction, which indicated the effectiveness of the base material reinforcement and confirmed the presence of hard phases in the structure. The width of the micro-traces of wear amounted to approximately 15 μm, whereas the average size of the sand grains used in the abrasive wear tests amounted to 250 μm. Detailed examination involving the use of confocal microscopy revealed the presence of cracks and single craters (having a depth of up to 250 μm) inside the layer (Figure 16b,d). The small ceramic particles uniformly distributed in the layer structure constituted an effective barrier to the grains of the abrasive. The abrasive wear process was intensified by the additional effect of loose particles of the hard phase torn out of the matrix and moving between the (interacting) surface of the specimen and that of the counterspecimen (“rubber wheel”). The aforesaid situation slightly increased the abrasive wear of the surface layer. The freely rolling sharp-edged particles of titanium carbide (TiC) were primarily responsible for the formation of micro-scratches on the counterspecimen surface or the plastic deformation of matrix fragments, manifested by characteristic micro-ridges. In publication [63] concerning their research work, the Authors also confirmed that abrasive wear was a dominant factor responsible for damage to the surface layer of Co-Cr-W-Mo alloys. In relation to data contained in previous publications [31] and individual research [30] concerning the abrasive wear resistance of surface layers made of cobalt alloys reinforced only with TiC particles, it was possible to notice the favourable abrasion-resistant effect of synthetic metal–diamond composite particles constituting approximately 20 wt% of the entire matrix reinforcement. In cases of abrasion-resistant cobalt-based alloys containing ceramic particles, abrasive wear resistance increased along with a growth in the volume fraction of the hard phase. It should be noted that under in situ conditions, the effect of natural factors including the ground structure, texture and presence of stresses, as well as hydrogeological conditions and humidity, abrasive wear resistance may differ from the test results presented in the article.

### 3.6. Impact Resistance Test Results

**Figure 17:**
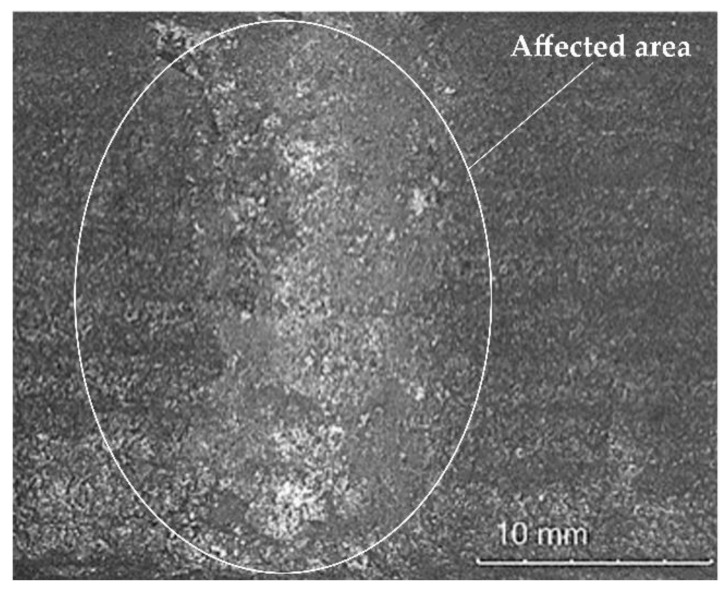
Macrostructure of the affected area. Condition of the surface of the composite layer (PPTAW metal deposition method, Co3+TiC+PD-W composite powder) deposited on structural low-alloy steel AISI 4715 after impact resistance tests.

The condition of the surface layer during the individual stages of impact resistance tests is presented in Figure 17.

The deposited composite layer was characterised by favourable resistance to moderate dynamic impact loads. After a cycle of twenty (ram) strokes affecting the surface of the layer with a potential energy of 200 J and the visual tests concerning the affected area, no damage in the form of visible cracks or chips of the weld deposit was observed. The only visible deformation was the plastic distortion of the deposited layer surface having a depth of approximately 0.3 mm, indicating the effective reinforcement of the cobalt-based alloy by cold work hardening.

## 4. Conclusions

The research-related tests aimed to assess the metallographic structure and to identify the metal-mineral abrasive wear resistance and the impact resistance of powder plasma transferred arc welding (PPTAW), which are innovative in terms of the chemical composition and the type of the hard phase reinforcing the matrix. The layer is intended for contact surfaces of inserts in drilling tools used in the extraction industry. The analysis of the above-presented test results justified the formulation of the following conclusions:

1. The chemical composition, type, amount and the size of the particles of the hard and metallic phases of the cobalt alloy-based composite powder enable its highly accurate and repeatable feeding, ensure its excellent melting and provide good weldability when using PPTAW metal deposition systems.
2. The cladding with powder plasma transferred arc welding (PPTAW) method (i.e., with the composite powder fed directly to the melt pool) favours the maintaining of the structural and thermal stability of the particles of the ceramic reinforcement of the matrix (having the form of tungsten-coated synthetic metal–diamond composite). During the solidification of the liquid metal in the melt pool, some of the reinforcement particles (TiC) underwent brittle cracking. The cracking process was probably triggered by the concentration of tensile stresses in carbide defects.
3. The composite layer was characterised by high hardness, very high metal-mineral abrasive wear resistance, relatively low internal porosity and advantageous resistance to moderate dynamic impact loads.
4. The mechanical and tribological features of the PPTAW deposited layer made using the innovative Co3+TiC+PD-W composite powder appear promising as regards the use of the layer on the contact surfaces of inserts in drilling tools applied in the used in the extraction industry.

The second part of the article will contain test results concerning the structural and the mechanical properties of a nickel-based layer reinforced with particles of tungsten carbide (WC) and synthetic metal–diamond composite.

## Figures and Tables

**Table 1:** Chemical composition of low-alloy structural steel AISI 4715 according to the manufacturer data (TimkenSteel Ltd., Canton, OH, USA).

Chemical Composition, wt.%								
C	Mn	S	P	Si	Cr	Mo	Ni	Fe
0.12–0.18	0.65–0.95	≤0.015	≤0.015	0.15–0.35	0.40–0.70	0.45–0.60	0.65–1.00	Bal.

**Table 2:** Chemical composition of Co3+TiC+PD-W powder.

Chemical Composition of Co 3 Alloy Matrix, wt.%									Ceramic Reinforcement of the Matrix, wt.%	
C	Si	Mn	Cr	Ni	Mo	W	Fe	Co	TiC	PD-W
2.5–3	≤1	≤2	24–28	≤3	≤1	12–14	<5	Bal.	90	10
Carbide-to-matrix ratio: 60/40 (wt.%)										

**Table 3:** Optimum processing parameters of robotic plasma powder transferred arc cladding of Co3+TiC+PD-W composite powder deposition on steel AISI 4715.

Process Parameters	Value of Parameter
Main arc current, I_a_ (A)	80
Pilot arc current, I_p_ (A)	15
Arc voltage, U (V)	25
Cladding speed, S (mm/s)	2.5
Powder feed rate, q (g/min)	15
Plasma gas flow rate, Q_p_ ^(1)^ (L/min)	1.6
Shielding gas flow rate, Q_o_ ^(2)^ (L/min)	12
Carrier gas flow rate, Q_s_ ^(2)^ (L/min)	4
Nozzle-workpiece distance, l (mm)	5
Overlap ratio, O (%)	33
Heat input, E_u_ ^(3)^ (J/mm)	480

Notes: ^(1)^ Argon 5.0 (99.999%) acc. ISO 14175—I1: 2009 was used as plasma, ^(2)^ argon/hydrogen 5% H2, Ar (welding mixture ISO 14175-R1-ArH-5) was used as shielding and carrier gas, ^(3)^ calculated acc. to the formula: E_u_ = k∙(U × I)/v The thermal efficiency coefficient for plasma transferred arc k = 0.6 was used.

**Table 4:** Results of the X-ray qualitative phase analysis of the surface layer (PPTAW metal deposition method, Co3+TiC+PD-W composite powder) deposited on structural low-alloy steel AISI 4715.

ICSD Card No.	Phase Name	Chemical Formula	Percentage (%)	Crystalline Structure
98-015-1365	Titanium carbide (1/1)	TiC	56.7	Regular (F m -3 m)
98-026-0166	Tungsten carbide (1/1)	WC/(PD-W) ^(1)^	11.9	Hexagonal (P -6 m 2)
98-062-2439	Cobalt	Co	31.4	Regular (F m -3 m)

**Table 5:** Results of the µCT analysis and calculations concerning the surface layer (PPTAW metal deposition method, Co3+TiC+PD-W composite powder) deposited on structural low-alloy steel AISI 4715.

Physical Quantity	Average Value of Measured Quantity
Density ρ (measured using the Archimedes method), g/cm^3^	5.7785
Absorbability A, %	1.0219
Open porosity P_o_, %	5.6467
Closed porosity P_c_, %	0.2526
Apparent density ρ_a_, g/cm^3^	5.4787
Total porosity P_c_, %	5.8993

**Table 6:** Hardness (HV10) measurement results concerning the external surface of the layer (PPTAW metal deposition method, Co3+TiC+PD-W composite powder) deposited on structural low-alloy steel AISI 4715 and of the surface of the reference material (abrasion-resistant steel AR400).

Hardnesess, (HV10)								
Specimen Designation	Specimen Number	Measurement Point Number					Average Hardness of the Tested Samples	Average Hardness of the Tested Materials
		1	2	3	4	5		
Co3+TiC+PD-W	C 01	673	733	657	733	657	690.6	688.7
	C 02	657	675	675	733	694	686.8	
AR400 Steel	S 01	430	421	420	429	424	424.8	424.1
	S 02	421	424	422	421	429	423.4	

**Table 7:** Results of the mineral-metal abrasive wear resistance tests concerning the surface layer (PPTAW metal deposition method, Co3+TiC+PD-W composite powder) deposited on structural low-alloy steel AISI 4715 in comparison with the abrasive wear resistance of abrasion-resistant steel AR400.

Specimen Designation	Spec. Number	Mass Before Test, g	Mass After Test, g	Mass Loss, g	Average Mass Loss, g	Material Density, g/cm^3^	Average Volume Loss, mm^3^	Relative ^(1)^ Abrasive Wear Resistance
Composite coating								
Co3+TiC+PD-W	C 01	149.7652	149.7560	0.0092	0.0093	5.7997	1.6035	139.64
	C 02	149.4113	149.4019	0.0094				
Reference material								
AR400 Steel	S 01	123.9290	122.2067	1.7223	1.7429	7.7836	223.9195	1
	S 02	121.7386	119.9752	1.7634				

Note: ^(1)^ relative abrasive wear resistance in relation to abrasion-resistant steel AR400.

## Data Availability

The data are not publicly available due to initiation of a patent procedure No. P435997.
